# Can a combination of different risk factors be correlated with leg fracture healing time?

**DOI:** 10.1007/s10195-012-0218-7

**Published:** 2012-11-22

**Authors:** Leo Massari, Francesco Falez, Vincenzo Lorusso, Giacomo Zanon, Luigi Ciolli, Filippo La Cava, Matteo Cadossi, Eugenio Chiarello, Francesca De Terlizzi, Stefania Setti, Francesco Maria Benazzo

**Affiliations:** 1 Orthopaedic and Traumatology Department, Sant’Anna Hospital, University of Ferrara, Via Savonarola 9, 44121 Ferrara, Italy; 2 Orthopaedic and Traumatology Department, S. Spirito in Sassia Hospital, Lungotevere in Sassia 1, 00193 Rome, Italy; 3 Orthopaedic and Traumatology Department, IRCCS Foundation San Matteo Hospital, University of Pavia, Viale Camillo Golgi 19, 27100 Pavia, Italy; 4 II Orthopaedic and Traumatology Clinic, Rizzoli Orthopaedic Institute, University of Bologna, Via Pupilli 1, 40134 Bologna, BO Italy; 5 IGEA SpA, Clinical Biophysics, Via Parmenide 10/A, 41012 Carpi, MO Italy

**Keywords:** Leg fracture, Delayed healing, Risk factor score

## Abstract

**Background:**

A multicenter retrospective analysis of patients treated for leg fractures was conducted to develop a score that correlates with fracture healing time and to identify the risk gradient for delayed healing.

**Methods:**

Fifty-three patients were analyzed and considered healed when full weight bearing was possible. Patients were divided into those who healed within 180 days and those who took longer to heal. Risk factors associated with delayed healing, fracture morphology, and orthopedic treatments were recorded. The available literature was used to weight the relative risk associated with each factor; values were combined into a score evaluating the risk of delayed healing: L-ARRCO (a literature-based score where the risk of delayed bone healing is calculated using a specific algorithm). Other risk factors associated with delayed healing were then considered in order to calculate a new score, ARRCO. Continuous variables were compared between groups using Student’s heteroschedastic two-tail *t* test. Receiver operating characteristic (ROC) curves and the areas under the curves were calculated to determine the ability of this score to discriminate subjects with delayed healing.

**Results:**

The mean L-ARRCO scores of the patients who healed within and after 180 days were significantly different (5.78 ± 1.59 and 7.05 ± 2.46, respectively). The mean ARRCO scores of the patients who healed within and after 180 days were also significantly different (5.92 ± 1.78 and 9.03 ± 2.79, respectively). However, the area under the ROC curve was significantly smaller for L-ARRCO than for ARRCO (0.62 ± 0.09 versus 0.82 ± 0.07).

**Conclusions:**

The ARRCO score is significantly associated with fracture healing time and could be used to identify “fractures at risk,” allowing early intervention to stimulate osteogenesis.

## Introduction

Fracture healing begins immediately after the traumatic event and continues until reconstitution of the mechanical competence of the bone is complete. For leg fractures, this process is normally completed within 3–4 months, but can take six months or longer [1–3]. It is estimated that 13 % of tibia fractures present delayed healing [4]. This complication involves a prolonged period of functional limitation and delayed rehabilitation, and can require surgery to reactivate and finalize the bone repair process.

Early identification of fractures that may be at risk of delayed healing would be advantageous to patients, and would allow early intervention, leading to a significant reduction in social health costs. Several clinical studies have identified a series of factors associated with the risk of prolonged healing time for fractures. However, no effective and reliable procedures exist for the early identification of a fracture at high risk of delayed healing. Identified risk factors are related to: (1) the patient’s clinical history, including age [5], gender [6, 7], smoking habit [6, 8, 9], diabetes [10], and alcoholism [11]; (2) the morphology of the fracture including the site [6, 7, 10, 12–14], diastasis of the stumps [15, 16], the presence of a skin lesion and its degree [15, 17–21], and the fracture mechanism [15, 22]; (3) the treatment of the fracture, including the synthesis device [4] and the duration of surgical intervention [4].

The majority of clinical studies have identified these risk factors using a univariate model of statistical analysis, but this does not take into account the interdependence of the factors considered. Attempts to assess the importance and interdependence of the various risk factors in a multivariate model are restricted to a small number of examples; the authors focus their analysis onto characteristics of the fracture and neglect patient history factors, as reported by Audigé et al. [4] in a retrospective study and by Hee et al. [5] in a prospective study. Nevertheless, there is currently no score that is calculated at the time of fracture which could provide an estimate of the time required for the fracture to heal or the gradient of risk that the fracture will result in delayed union.

In this study, clinical records from patients treated for leg fractures were reviewed to collect detailed information concerning patient history risk factors, fracture morphology, and orthopedic treatment. Various factors were combined in the ARRCO score (where the risk of delayed bone healing is calculated using a specific algorithm) as proof of the principle that a score can be correlated with healing time.

## Materials and methods

### Patients

Ninety-three patients treated for leg fractures were analyzed retrospectively between 2007 and 2009 at three orthopedic centers: the Orthopedic and Traumatology Department, Sant’Anna Hospital, University of Ferrara; the Orthopedic and Traumatology Department, IRCCS Foundation San Matteo Hospital, University of Pavia; the Orthopedic and Traumatology Department, Santo Spirito in Sassia Hospital, Rome.

Inclusion criteria were: signed informed consent to collect clinical data; leg fractures treated conservatively and/or surgically. Exclusion criteria were: osteoporosis; leg fractures involving the tibial plateau; and isolated fractures of the malleolus.

The study was authorized by the local ethical committees and was performed in accordance with the ethical standards of the 1964 Declaration of Helsinki as revised in 2000.

### Data collection

Data contained in each patient’s record were recorded in an electronic case report form (e-CRF) that was created especially for this study and developed using object-oriented C# programming and the Microsoft.Net 2.0 Framework. Table 1 presents the information collected on patient history risk factors and the morphology and treatment of the fracture.

**Table 1:** Patient risk factors

Patient history factors	Age; sex; height; weight; smoking status (amount and since when); diabetes; malnutrition; abuse (alcohol, narcotics, etc.); drugs used (antibiotics, NSAIDs, corticosteroids, other, specifying the active principle and dosage); associated pathologies; previous surgery
Fracture morphology	Date of trauma; site (tibia, fibia, tibia and fibia), level (proximal or distal), and lesion zone (epiphysis, metaphysis, diaphysis); side of fracture (right or left); AO classification; type of trauma (high/low energy, details on origin of trauma); type of fracture (closed, exposed <5 cm, exposed >5 cm, open grade I); loss of bone (and details); associated lesions (cutaneous, nervous, tendon, muscular, vascular, none, others); blood loss and hemoglobin value in blood; previous interventions at lesion site (and details); presence of synthesis device at time of trauma; alignment, stability and diastasis between stumps (2, 4, 6, 8, 10 mm) before treatment
Treatment of fracture	Date of orthopedic treatment; treatment (surgical or conservative); conservative treatment details (cast, brace, other); surgical treatment details (mini-invasive surgery); synthesis device (external fixator, endomedullary nail, plate, locking compression plate, other); further treatment for stabilization (cast, brace, other, none); treatment with autologous bone grafts, homoplastic grafts, stem cells, mesenchymal cells, saw bone, platelet gel, other, none; length of surgery (<200 or >200 min); intraoperative complications (cutaneous, nervous, tendon, vascular, bone, none, other); blood loss and hemoglobin count; complications immediately after operation (24 h); administration of drugs after treatment (antibiotics, NSAIDs, corticosteroids, other, specifying active principle and dosage); thrombo-embolism prophylaxis; alignment, stability and minimum diastasis between stumps (2, 4, 6, 8, 10 mm) after treatment; biophysical therapy (details, start date) at follow-up; drugs used at follow-up (antibiotics, NSAIDs, corticosteroids, other, specifying active principle and dosage); infection at follow-up; removal of fixing device at follow-up; removal of fixing device at follow-up; new treatment (surgical or conservative) at follow-up at the lesion site; re-fracture at follow-up at the lesion site; alignment, stability and minimum diastasis between stumps (2, 4, 6, 8, 10 mm) at follow-up; clinical healing at follow-up (patient has no functional limitation)

Table lists the data collected concerning the risk factors for patients selected during 2007–2009 at the three orthopedic centers enrolled in the study

As this was a retrospective study, X-ray controls were not always available. Therefore, clinical healing of the patient was considered the end-point. Patients were classed as healed when there was no further limitation to limb function and no further radiographic or clinical controls were required to confirm effective healing of the fracture. Patients were considered clinically healed when full weight-bearing was allowed without support and pain.

L-ARRCO was calculated, where L indicates the exclusive employment of parameters identified in the literature as being associated with prolonged healing time. Each of the parameters was assigned a score ranging from zero to four as a function of the RR (relative risk) value [4, 5, 17, 18], as demonstrated in Table 2. The L-ARRCO score reached a maximum of 20 and was the sum of the scores assigned to each risk factor.

**Table 2:** Risk factors used for the L-ARRCO score

Risk factor	L-ARRCO score
Patient history factors	
Age	
<46	1
46–60	2
>60	3
Obesity	1
Smoking status	1
Use of NSAID	1
Use of steroids	1
Diabetes	1
Fracture morphology and orthopedic treatment	
Type of fracture	
Closed	1
Exposed < 5 cm	2
Exposed > 5 cm	3
Open grade I	4
Localization	
Diaphysis	1
Epiphysis-metaphysis	2
Treatment	
Conservative	0
Plate	1
Endomedullary nail	2
External fixator	3
Alignment	1
Stability	1
Diastasis	1

Table provides a description of the risk factors used to calculate the L-ARRCO score

In a second analysis, all parameters collected in the e-CRF were considered and their association with prolonged healing time was calculated using logistic analysis (RR). A second algorithm, ARRCO, whose values ranged from 0 to 26, was calculated.

### Statistical analysis (level of significance set at *p* < 0.05)

Statistical analysis was performed using SPSS 15.0 software (SPSS Inc., Chicago, IL, USA). The characteristics of the population analyzed were described by calculating the mean value, standard deviation, and maximum and minimum values.

To conduct univariate logistic analysis to determine the RRs, delayed healing was attributed to a patient who had not attained clinical healing at 180 days, as previously defined.

The correlation between score and healing time was calculated using the linear regression test and Spearman’s correlation coefficient. Continuous variables were compared between the two groups using Student’s heteroschedastic two-tail *t* test.

Analysis by ROC (receiver operating characteristic) curve and calculation of the area under the curve (AUC) was used to determine the ability of the score to discriminate subjects with delayed healing from others. Each point on the curve represents a threshold value of the analyzed score for which the sensitivity and specificity can be calculated. The sensitivity of the test is the percentage of pathological subjects correctly identified by the method with respect to the whole group of pathological subjects; the specificity is the percentage of healthy subjects correctly identified as healthy with respect to the group of effectively healthy subjects.

## Results

Of the 93 patients, the information required to complete the e-CRF was available for 53 individuals (38 male, 15 female). The characteristics of these subjects are summarized in Table 3. For 47 patients, the fracture was treated with a single surgical operation; the remaining patients underwent a second operation.

**Table 3:** Patient characteristics

	Mean	SD	Min	Max
Age (years)	46.4	21.3	17	95
Height (cm)	171	8	150	188
Weight (kg)	71.9	13.4	30	105
BMI (kg/m^2^)	24.6	3.7	13	33

Table shows anagraphic and anthropometric data for the 53 subjects considered

### Analysis of correlation with healing time

**Fig. 1:**
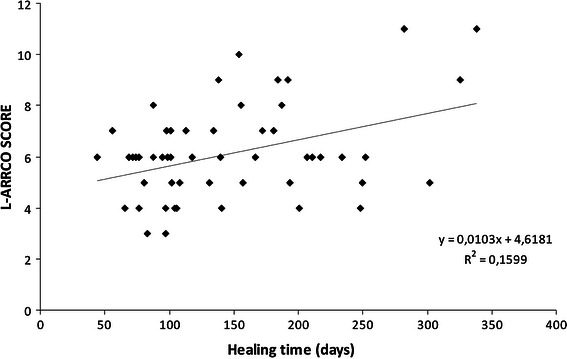
Analysis of the correlation between clinical healing time and L-ARRCO score

The L-ARRCO score was calculated for each patient. The Pearson coefficient of correlation between the L-ARRCO score and clinical healing time was positive, with a value *r* = +0.400, *p* = 0.003 (Fig. 1).

**Fig. 2:**
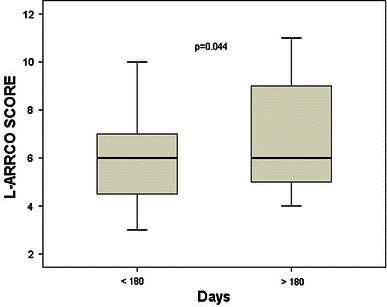
Box plot of the L-ARRCO scores for the two groups analyzed: <180 days (patient healed), >180 days (patient suffered delayed healing). The *horizontal line* *in thebox* indicates the median, the *box* indicates the standard deviation, and the *vertical bars* indicate the confidence interval at 95 %. The *p* *value* indicates the comparison between the two groups using Student’s *t* test

In the group of patients analyzed, 36 attained clinical healing within 180 days, and 17 were defined as patients with delayed healing. The mean value of the L-ARRCO score for patients who healed within 180 days was 5.78 ± 1.59 (CI 5.24–6.31), and that for the patients who healed after 180 days was 7.05 ± 2.46 (CI 5.73–8.03), *p* = 0.044. Figure 2 shows the score distributions for the two groups. This first analysis demonstrates that patients with an L-ARRCO score of between four and six had healing times ranging from 44 to 302 days. Therefore, the analysis was focused on these groups of patients to identify other risk factors not included in the L-ARRCO score that limit the specificity and sensitivity of the score.

**Fig. 3:**
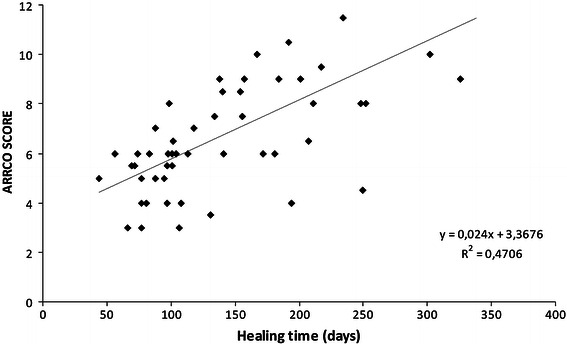
Analysis of the correlation between clinical healing time and ARRCO score

Using the univariate logistic model, factors with a significant relative risk value, *p* = 0.05, were identified (Table 4). These factors were used to calculate the ARRCO score for each patient. The linear correlation between the ARRCO score and the clinical healing time was analyzed (Fig. 3). The Pearson correlation coefficient was positive, +0.690, *p* < 0.0001—significantly higher than that previously obtained (*p* < 0.0001).

**Table 4:** Parameters associated with prolonged healing time

Parameter	Relative risk	Confidence interval 95 %
Malnutrition	4.1	1.3–48.4
Tibia fracture without fibia involvement	1.6	1.0–4.8
Loss of bone substance	4.0	1.1–15.8
Graft with saw bone	12.3	1.3–114.6
Plate + diastasis	6.0	1.0–34.4
Plate + instability	3.2	1.0–20.8
Locking compression plate	2.8	1.2–10.8
Plate + blood loss	4.4	1.2–16.1
Plate + plaster^a^	0.9	0.3–1.0
Age (age classes)	1.2	1.0–2.3
Obesity	1.5	1.1–7.5
Smoking	3.0	1.4–9.9
Type of fracture (closed; exposed < 5 cm; exposed > 5 cm, open grade I)	2.0	1.1–3.8
Localization (diaphysis; epiphysis-metaphysis)	3.1	1.3–10.0
Instability	1.8	1.1–5.5
Diastasis	1.4	1.0–4.2
Alignment^a^	0.4	0.0–0.8
Treatment (conservative; plate; endomedullary nail; external fixator)	7.9	2.5–25.3

Table shows parameters associated with prolonged healing time

^a^Associated with reduced risk

**Fig. 4:**
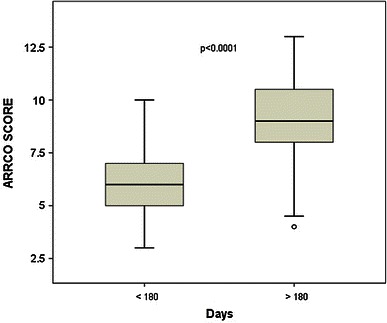
Box plot of the ARRCO scores for the two groups analyzed: <180 days (patient healed), >180 days (patient suffered delayed healing). The *horizontal line* *in thebox* indicates the median, the *box* indicates the standard deviation, and the *vertical bars* indicate the confidence interval at 95 %. The *p* *value* indicates the comparison between the two groups using Student’s *t* test

The mean value of the ARRCO score for patients who healed within 180 days was 5.92 ± 1.78 (CI 5.31–6.52), and that for patients who healed after 180 days was 9.03 ± 2.79 (CI 7.55–10.33), *p* < 0.0001 (Fig. 4).

**Fig. 5:**
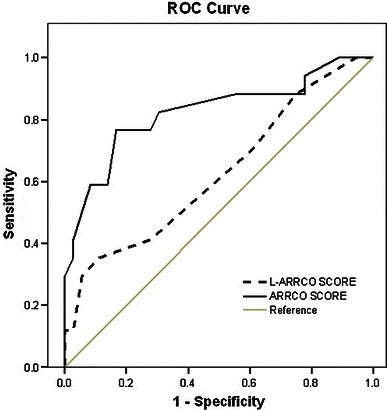
ROC curve for discriminating subjects with healing times of <180 days from subjects who suffered delayed healing

In the discrimination analysis between subjects who healed within 180 days and those who took longer than 180 days, the ROC curve with the ARRCO score gave an AUC that was significantly greater (0.82 ± 0.07, CI 0.69–0.96) than that obtained with the L-ARRCO score (0.62 ± 0.09, CI 0.46–0.79), *p* < 0.0001 (Fig. 5). Importantly, for 70 % specificity values, a sensitivity of 82 % was achieved with the ARRCO score, whereas only 41 % sensitivity was achieved with the L-ARRCO score.

## Discussion

It is difficult to assess whether, and with what probability, a fracture will evolve into delayed union or a failed union, often preventing early action that could be taken to enhance healing. It is only a posteriori (i.e., after the onset of the complication) that evidence of a series of risk factors that could not be immediately identified at the time of trauma or immediately following treatment (whether surgical or conservative) can be identified. There are no reliable clinical or laboratory investigations that can identify “fractures at risk” (so-called because they require a prolonged time to heal).

Various studies have demonstrated that a high-energy trauma, loss of bone and cutaneous substance, associated nerve and vascular lesions, the co-presence of diseases such as diabetes, and smoking are all factors that contribute to prolonged healing time of a fracture [4]. The associated RR has been calculated for each of these factors, but they have not been combined to obtain a risk gradient with good sensitivity and specificity that could find valid clinical application [4, 5, 17, 18].

In the present work, the L-ARRCO score was developed by combining the parameters reported in the literature [4–8, 10–22] and tested on a group of patients treated for leg fractures to assess its correlation with healing time. The decision to confine this investigation to the leg alone was based on the following considerations: leg fractures are very common, and excluding those due to bone fragility from osteoporosis ensure that the frequency of delayed healing is sufficiently high to provide concrete data. Furthermore, considering more fracture sites would have involved a significant increase in the number of variables and patients analyzed.

The L-ARRCO values were correlated with healing time (*r* = +0.40); however, the sensitivity of the score was not satisfactory, as although a significant number of patients obtained a low score, their healing times showed an extremely wide range. In fact, the ROC curve gave an AUC that was not particularly high (0.62 ± 0.09), which implies that the sensitivity and specificity of the score would not be satisfactory at any point on the curve. A cluster of patients with scores ranging between four and six who took longer than 180 days to heal were responsible for this low sensitivity.

However, data collected from the patients’ hospital records allowed other parameters associated with delayed healing to be identified and used in a second score, named ARRCO. Satisfactory values of sensitivity (82 %) and specificity (70 %) were obtained with ARRCO. In addition, the correlation of the ARRCO score values with fracture healing time (*r* = +0.69) was significantly better than seen for the previous score.

The main limitation of this study is that it is a retrospective analysis and includes a limited number of patients. However, this study clearly demonstrates, as a proof of principle, that a complete and balanced evaluation of the various risks present at the time of fracture can reliably identify the majority of the patients who may suffer prolonged healing times.

This study has identified relevant information—clinical and associated with surgery—that should be collected in a prospective study. Using power analysis, it has been calculated that the number of patients required for such a prospective study would be 300. Performing such a prospective study would lead to the identification of multiple conditions that influence the process of fracture healing, alone and in combination, and would allow their importance to be assessed. Therefore, a reliable score to estimate the risk gradient for prolonged fracture healing time could be developed.

The software available could easily be adapted and used in orthopedic practice. After reliably calculating the predicted risk of delayed healing, the orthopedic surgeon could prescribe therapy that can be applied earlier than is currently the case, for example favoring osteogenetic activity using systemic or local drug therapy, or by stimulating the endogenous synthesis of bone morphogenetic proteins using physical stimuli. The identification and selection of fractures at risk using ARRCO also represents an important study tool, as it makes it possible to test and focus the study of new therapeutic interventions on a limited but specific number of patients, as well as to assess treatment costs for fractures at risk.
